# TRENDS IN US NURSING HOMES RESIDENTS WITH LIMITED ENGLISH PROFICIENCY (LEP)

**DOI:** 10.1093/geroni/igae098.3176

**Published:** 2024-12-31

**Authors:** Maureen Mickus, Jade Nguyen

**Affiliations:** Western Michigan University, Kalamazoo, Michigan, United States; Western MIchigan University, Kalamazoo, Michigan, United States

## Abstract

Ethnic elders residing in U.S. nursing homes experience significantly lower quality of life compared to their white counterparts. Among the most vulnerable are those with Limited English Proficiency (LEP) who may be unable to communicate even basic needs to staff. Based on the Minimum Data Set (MDS), this study examined the prevalence of LEP residents in the U.S. between 2020 and 2023 as defined by those needing language interpreter services. From 2020 to 2021, the number of LEP residents decreased by 25.3% from 46,877 to 35,021, in part due to the disproportionate rate of death from COVID-10 among minorities. After 2021 an increase was reported by an estimated 2,000 residents per year with an estimated 39,584 LEP residents recorded in 2023. The need for interpreter services in nursing homes varied significantly by state with 14.4% of California and 7.71% of New York residents as compared with 14 states reporting less than 1%. Not surprisingly, U.S. Census statewide data of individuals age 65+ in the general population reporting speaking English less than “very well” was positively correlated with the percentage of LEP residents in nursing facilities, r (47) =.91, p <.001. Based on the unmet needs that are likely associated with LEP residents and the unavailability of interpreters 24/7 in facilities, increased cultural education and sensitivity is warranted for staff in long-term care facilities. Future research determining which specific languages are spoken in facilities in addition to ways to improve communication is needed.

